# Segmentation-Based Blood Blurring: Examining Eye-Response Differences in Gory Video Viewing

**DOI:** 10.3390/s25072093

**Published:** 2025-03-27

**Authors:** Jiwon Son, Minjeong Cha, Sangkeun Park

**Affiliations:** Department of Software Convergence, Kyung Hee University, Yongin 17104, Republic of Korea; sjw0592@khu.ac.kr (J.S.); minjeongcha@khu.ac.kr (M.C.)

**Keywords:** viewing experience, segmentation-based blurring, gory video, eye tracking, physiological responses, HCI

## Abstract

Online video platforms have enabled unprecedented access to diverse content, but minors and other vulnerable viewers can also be exposed to highly graphic or violent materials. This study addresses the need for a nuanced method of filtering gore by developing a segmentation-based approach that selectively blurs blood. We recruited 37 participants to watch both blurred and unblurred versions of five gory video clips. Eye-based physiological and gaze data, including eye openness ratio, blink frequency, and eye fixations, were recorded via a webcam and eye tracker. Our results demonstrate that partial blood blurring substantially lowers perceived gore in more brutal scenes. Additionally, participants exhibited distinctive physiological reactions when viewing clips with higher gore, such as decreased eye openness and more frequent blinking. Notably, individuals with a stronger fear of blood showed an even greater tendency to blink, suggesting that personal sensitivities shape responses to graphic content. These findings highlight the potential of segmentation-based blurring as a balanced content moderation strategy, reducing distress without fully eliminating narrative details. By allowing users to remain informed while minimizing discomfort, this approach could prove valuable for video streaming services seeking to accommodate diverse viewer preferences and safeguard vulnerable audiences.

## 1. Introduction

With the expansion of internet infrastructure, more people than ever have access to high-speed online services [1,2]. This increased connectivity has fueled the global popularity of streaming platforms such as YouTube and Over-The-Top (OTT) media services (e.g., Netflix). According to a Statista report, approximately 3.1 billion OTT video mobile apps were downloaded worldwide as of 2023 [3]. Short-form video platforms like YouTube Shorts, TikTok, and Reels have also shown rapid growth, highlighting the pervasiveness of online video consumption. As smartphones and mobile networks become widespread, video streaming has evolved into a daily activity for a broad spectrum of users.

Despite the convenience and entertainment value of online videos, harmful or inappropriate material—such as gory or violent clips—remains easily accessible to minors with minimal oversight. For instance, social media platforms like Instagram, Snapchat, and TikTok can expose children and adolescents to unsuitable content [4,5], and a large-scale Mozilla Foundation study found that approximately 15% of user “regrets” on YouTube were due to violent or graphic content recommended by the platform [6].

Viewers are also becoming desensitized to violent imagery, often seeking more intense stimuli for continued engagement [7]. This issue is amplified by the ease with which anyone—including children—can encounter disturbing scenes [8,9]. Moreover, prolonged exposure to violent media correlates with increased aggression and hostility [10,11], and unexpected encounters with blood or gore can trigger strong disgust or revulsion [12]. These findings underscore the need for targeted solutions that limit exposure to graphic content without eliminating its informative or contextual elements.

Many studies have explored automated techniques to detect and filter explicit or harmful video content (e.g., violence or nudity) through machine learning and deep learning [9,13,14]. Some methods block or warn about flagged segments [8,15], while others remove violent frames entirely [16] or apply a blurring technique to obscure inappropriate scenes or objects [17,18,19,20,21]. Although these strategies effectively limit audiences’ exposure to problematic content, most research focuses on detection accuracy and filtering performance, neglecting to examine how such interventions influence user experiences.

Studies on viewers’ physiological and behavioral responses to gruesome or frightening material have revealed that variables like blink frequency, eye gaze, and heart rate shift when exposed to violent imagery [22,23,24,25]. However, many of these works compare highly disturbing stimuli with entirely neutral clips, leaving a gap in understanding how partial reductions in gore—such as segmentation-based blurring—affect the viewing experience. Additionally, individual differences in fear or phobia may significantly alter users’ reactions, including avoidance behavior and elevated arousal [26,27,28].

To address these gaps, we developed a segmentation-based blood-blurring model aimed at reducing the intensity of violent scenes. We then compared user experiences when watching blurred versus unblurred versions of the same clips. Using a webcam and an eye tracker, we collected eye-focused physiological and gaze data—*Eye Openness Ratio*, *Blink Frequency*, and *Eye Fixations*—to answer the following research questions:

- RQ1: To what extent does a segmentation-based blood blurring reduce viewers’ perceived gore?
- RQ2: How do different levels of gore influence viewing experience (i.e., eye-focused physiological reactions and eye gaze)?
- RQ3: In what ways is an individual’s fear level (i.e., blood- or injury-related phobias) associated with the viewing experience of gory videos?

Through an experiment involving 37 participants, we observed that (1) our blood-blurring model effectively lowers perceived gore, (2) participants exhibit significant differences in physiological and attentional indicators when viewing more graphic footage, and (3) an individual’s fear of blood (particularly blood-self) correlates with increased blink frequency.

Our study makes several contributions to Human–Computer Interaction (HCI). First, it transcends binary comparisons of highly violent versus neutral content by investigating how varying levels of gore affect viewer experience. Second, by monitoring physiological signals and gaze patterns, it shows that partial blurring can mitigate distress without fully removing underlying details. Third, these insights inform design implications for interactive video platforms, such as adjustable blood blurring or responsive moderation aligned with personal gore tolerance. Ultimately, our work supports more nuanced content moderation strategies, helping viewers stay informed without unnecessary exposure to graphic imagery.

## 2. Related Work

### 2.1. Detection and Filtering of Inappropriate Video Content

A significant body of research has focused on detecting and filtering inappropriate or harmful content in videos, such as violence and nudity, using machine learning and deep learning approaches. For instance, researchers have proposed methods that analyze visual cues (e.g., bloodstains and weapons) and auditory signals (e.g., gunshots and screams) to identify violent scenes [9,13]. Others have expanded these techniques to handle multiple modalities, including text, images, and video, in order to recognize not only violence or sexual content but also hate speech [14].

Beyond mere detection, several studies focus on warning users or completely blocking access to content deemed inappropriate. One system [8] issues alerts to a guardian whenever TikTok detects violent or unethical clips, allowing the guardian to restrict access. Similarly, other approaches filter or remove flagged segments, effectively preventing underage users from encountering explicit video content [15]. Some systems even delete frames containing violent scenes to reconstruct clean versions of the content [16].

Another line of research focuses on using blurring techniques to hide explicit or graphic visuals in a video rather than blocking the entire content. Some methods identify entire scenes that are considered inappropriate or violent and apply a blur to these sections [17,18]. Other advanced approaches concentrate on detecting harmful or explicit objects in images or videos [19,20,21], using machine learning or deep learning to locate the problematic areas and obscure them without affecting the rest of the scene.

Although these strategies effectively limit the audience’s exposure to inappropriate or harmful content, most studies concentrate on detection accuracy and filtering performance without thoroughly examining how such measures affect the user’s viewing experience. Our study not only develops a deep learning model to detect harmful content in violent scenes and apply blurring but also explores how this intervention shapes viewers’ experiences. By examining physiological data (i.e., eye openness ratio and blink frequency), behavioral data (i.e., eye gaze), and self-reported (i.e., perceived gore) data, we seek to clarify the impact of blurring on audience reactions. This user-centered perspective provides valuable insights for developing advanced content moderation systems that balance user safety with a nuanced, customizable viewing experience.

### 2.2. Behavioral and Physiological Reactions to Inappropriate or Harmful Content

Research suggests that individuals exhibit distinctive facial reactions, including eye squeezing, when viewing inappropriate or harmful content. One study [29] reported more pronounced facial muscle tension, such as furrowed brows, among participants watching fear-related stimuli (e.g., accident scenes, aggressive animals) compared to those observing neutral images. Another investigation [25] found that viewers cringed at disgust-inducing images (e.g., spoiled food, contaminated objects).

In addition, several works [22,23,30] noted that blink frequency often rises in response to violent or gore-heavy imagery, possibly reflecting a heightened startle reflex. Further evidence indicates that participants blinked more often when viewing aggressive images involving explosions, gunshots, or violence, suggesting that heightened aggression can amplify bodily responses [31]. Another work discovered that blink frequency increased when viewing aggressive video clips that elicited disgust, reflecting both attention diversion and an emotional avoidance response [24].

Many viewers avert their gaze more swiftly when confronted with threatening or repulsive content. Researchers [25,28] observed that participants showed shortened fixation times and a faster tendency to avoid frightening stimuli compared to neutral scenes.

Heart rate, skin conductance, blood pressure, and respiration can also shift considerably during the viewing of gruesome or frightening material. Studies [11,22,23,32,33,34,35] discovered increased heart rate in response to fear-inducing clips, violent scenes, or the presence of blood. Electrodermal Activity (EDA), the electrical changes measured on the surface of the skin that result from the activation of sweat glands, often triggered by emotional or physiological arousal, tends to rise when individuals see images of snakes, open wounds, or realistic blood colors [22,32,33,36], signaling heightened autonomic arousal. Research also links blood phobia to higher blood pressure [27], and multiple experiments reported accelerated breathing rates among those confronted with frightening film clips or surgeries [27,32,33].

Most previous work compares physiological responses to highly disturbing content or entirely neutral material. These comparisons do not always capture how partially reducing gore influences audience reactions. Our study addresses these gaps by examining user responses to the same video clips presented in both blurred and unblurred formats. This design clarifies how decreasing the visibility of graphic elements affects blinking, gaze, and other measurements, and extends beyond mere avoidance to investigate whether participants actually look at or ignore the gruesome objects in both versions of the same clip.

### 2.3. Viewing Experiences Depending on Individual Characteristics

Several studies have examined how personal fears or phobias influence responses to gory or disturbing content. Researchers have classified participants into groups based on blood–injection–injury (BII) or blood phobia, and then compared their reactions when viewing surgical footage or other graphic stimuli [26,27]. Results showed that individuals with higher blood-related fears reported stronger anxiety and displayed elevated physiological arousal, such as increased heart rate, blood pressure, or heightened avoidance behaviors.

Other investigations have focused on variations in fear responses at the individual level rather than within predefined phobia groups. Some studies relied on validated scales such as the Fear Survey Schedule (FSS) [37], the Fear of Pain Questionnaire (FPQ) [38], and the Fear Questionnaire (FQ) [39] to assess each participant’s fear levels [28,30]. Higher fear ratings correlated with stronger physiological reactions, such as more frequent blinking, faster breathing, or a tendency to look away when viewing gory or threatening scenes. In a virtual reality setting, greater intensity of frightening stimuli was associated with more pronounced gaze aversion, an effect that was magnified among participants who scored high on fear questionnaires [28].

Many previous studies have compared videos featuring intense or explicit content (e.g., violence, gore, or other disturbing material) with entirely neutral or nonviolent footage. This approach leaves a gap in our understanding of how partial reductions in gore might influence viewers. To address this gap, our study presents the same clips in both blurred and unblurred forms, and then measures physiological and gaze-related responses. In addition, fear of blood is assessed through subscales that distinguish between blood-self and blood-other, allowing for a deeper analysis of how individual blood-related sensitivities interact with different levels of gore in the same video.

## 3. Methodology

### 3.1. Development of a Blood Detection and Blurring Algorithm

In this study, we aim to detect blood in video content and apply blurring to enhance viewers’ overall watching experience. To achieve this, we develop and evaluate a model capable of segmenting and blurring blood regions in video frames.

For blood detection in videos, we adopt YOLO-based instance segmentation models. YOLO is known for its rapid inference speed and high accuracy. Among the available models, we focused on YOLOv5s-seg and YOLOv8s-seg. YOLO offers various model sizes—nano (n), small (s), medium (m), large (l), and xlarge (x)—with different levels of complexity. We selected the small (s) models to balance performance and inference speed.

To fine-tune the YOLO models to detect blood, we utilized three open blood-segmentation datasets [40,41,42]. In total, 4787 images (and their corresponding annotations) were used. These images included various red objects—such as bloodstained items (e.g., knives, tissues), bleeding faces and body parts, injuries, gore movie scenes, surgical images, blood droplets, and specimen containers/syringes holding blood—as well as non-blood images (e.g., red carpets, red clothes/accessories, red fruits, red text, general faces, and objects). The annotation files contained segmentation boundary coordinates for all visible blood regions in each image. We trained instance segmentation models on the datasets described above, focusing on YOLOv5s-seg and YOLOv8s-seg. The hyperparameters for both models were set as follows: epoch = 50 or 100, batch = 64, and imgsz = 640.

To evaluate model performance, we performed 5-fold cross-validation. We used evaluation metrics, such as Precision, Recall, and Mean Average Precision (mAP). The evaluation result is shown in Table 1. The mAP metric provides a comprehensive assessment of detection performance by computing precision at various recall levels. Specifically, we adopted the two following variations of mAP [43]:

- *mAP50*: This version considers a true positive if the predicted and ground-truth bounding boxes have an Intersection over Union (IoU) of at least 0.5. Since a 50% overlap is relatively lenient, this metric can be viewed as a less strict standard of detection.
- *mAP50-95*: Here, IoU thresholds range from 0.50 to 0.95 in increments of 0.05. Each threshold’s mAP score is calculated, and these scores are then averaged. This approach is more stringent, requiring a larger overlap between predicted and ground-truth boxes than the previous criterion.

**Table 1:** Comparison of YOLO-based blood detection via instance segmentation: bounding boxes vs. segmentation masks.

Metric	YOLOv5s-Seg		YOLOv8s-Seg	
	Epoch = 50	Epoch = 100	Epoch = 50	Epoch = 100
Box				
Precision	0.825	0.869	0.787	0.837
Recall	0.695	0.732	0.633	0.691
mAP50	0.744	0.776	0.703	0.757
mAP50-95	0.498	0.555	0.503	0.578
Mask				
Precision	0.789	0.828	0.764	0.819
Recall	0.647	0.683	0.605	0.655
mAP50	0.688	0.726	0.661	0.718
mAP50–95	0.377	0.432	0.388	0.452

By comparing both mAP50 and mAP50-95, we can gauge not only the overall detection ability of our models but also how they perform under increasingly strict IoU requirements. The results demonstrate the effectiveness of our YOLO-based models in detecting blood across diverse scenarios. The evaluation result shows that the YOLOv8s-seg model trained for 100 epochs exhibited the most balanced performance in blood segmentation, making it our chosen model for subsequent analyses.

Using the chosen YOLOv8s-seg model, we applied *ffmpeg* [44] to extract the audio track and split the video into individual frames at a rate of 30 fps. Next, we used *cv2* [45] to read each frame and apply instance segmentation of blood. This process generated a pixel-wise segmentation map that assigned each pixel to a detected object instance, providing both *box coordinates* (for bounding boxes) and *mask coordinates* (for the segmented regions). The box coordinates, stored in a separate file along with frame numbers, were later leveraged for gaze-tracking experiments. The mask coordinates, which capture precise pixel boundaries, enabled the application of blur to identified blood regions. We then re-rendered each processed frame, combined it with the previously extracted audio track, and produced a final output video whose blood content was blurred.

### 3.2. Recruitment

Between December 2024 and January 2025, we conducted an experiment to examine the effectiveness of blurring blood scenes. A recruitment poster was created and distributed via the university’s online communities and bulletin boards, inviting potential participants to complete a screening survey. This survey, administered through Google Forms, included the adapted version of the Multidimensional Blood/Injury Phobia Inventory (MBPI) [46]. We adapted three blood-related subscales, *Blood-self* (4 items), *Injury* (6 items), and *Blood/injury-Others* (5 items) from the MBPI. The participants answered each item on a 4-point Likert scale (1 = very slightly or not at all; 4 = extremely). In addition, the survey also included sample images and video materials containing blood that participants would later view during the experiment. We recruited individuals who completed the survey and indicated they had no issues watching videos containing blood.

A total of 50 individuals initially indicated both their willingness to participate and their comfort with watching gory videos via the survey. However, 13 failed to schedule a visit or did not attend due to personal reasons (e.g., flu or scheduling conflicts), leaving 37 final participants (15 females; age: M = 24.7, SD = 2.0). This study was conducted after obtaining approval from the Institutional Review Board (IRB) (approval number: KHGIRB-24-701). All participants were fully informed about the study’s objectives, procedures, and data protection measures, and they voluntarily consented to participate. They also agreed to the collection and use of their data for research purposes and were informed of their right to withdraw at any time.

### 3.3. Experiment

Each participant was invited individually to a laboratory that was fully set up for the experiment. The authors were present to provide and explain the research guidelines. During the experiment, only one participant and the authors remained in the laboratory, and no one else was permitted to enter or leave, ensuring that participants could concentrate on the experiment.

As a preview, participants first watched a 1.5 min video clip where a soldier, shot in the abdomen during war, receives treatment, from *Saving Private Ryan (1998)* (01:28:45–01:30:15), to remind them that the experiment would involve scenes containing this kind of blood and gore. They were then asked to sign the consent form before proceeding with the main part of the experiment. Five primary video clips were used:

Clip (1) *Parasite (2019)*: A South Korean black comedy–thriller film directed by Bong Joon-ho. This clip includes a scene in which a knife murder turns a party into complete chaos. The video clip (00:00:02–00:02:03) is from YouTube [47].
Clip (2) *Dr. Romantic (2016)*: A South Korean television medical melodrama. This clip shows an emergency patient undergoing abdominal surgery in a hospital. The clip (00:03:44–00:08:02) is from YouTube [48].
Clip (3) *Nasal Tip Plasty Surgery*: The clip is about a cosmetic surgical scene. The clip (00:00:11–00:00:47) is from YouTube [49].
Clip (4) *The Witch: Part 2 (2022)*: A South Korean science fiction action horror film by Park Hoon-jung. This clip shows bullets being removed one by one from the body of a person lying as if dead. The clip (00:31:27–00:32:40) is from *Naver Series On*, which is an online VOD service [50].
Clip (5) *The Revenant (2015)*: An American epic Western action drama film directed by Alejandro G. Iñárritu. This clip shows a group of passersby discovering a person who collapsed after fighting a bear and administering emergency treatment. The clip (00:29:23–00:32:29) is from *Naver Series On*, which is an online VOD service.

All five video clips feature bloody scenes that might require blurring for some viewers. Each clip was provided in two versions (original and blurred), resulting in a total of 10 clips. Although the chosen clips differ in duration, we retained each clip’s natural length to preserve important contextual information because the contextual information strongly shapes perceived gore. Our repeated-measures design, where each participant viewed both the original and blurred versions of the same clip, mitigates potential effects from varying run times. To minimize learning effects, each participant viewed all 10 video clips in a randomized, counter-balanced order, accounting for both the clip sequence and the version type.

While each participant watched the videos, we collected three sensor-based measurements—**Eye Openness Ratio**, **Blink Frequency**, and **Gaze Coordinates**—using a webcam and an eye tracker. Therefore, prior to watching the short clip from *Saving Private Ryan (1998)*, each participant underwent an individual calibration session to enable precise tracking of gaze coordinates and eye openness.

**Eye Openness Ratio**: We used the Eye Aspect Ratio (EAR) algorithm [51] to track the degree of eye openness. EAR is a computer vision technique that measures how open or closed a person’s eyes are by tracking specific facial landmarks around each eye. The algorithm computes a ratio of the vertical distances between the eyelids to the horizontal distance across the eye: when the eyes are more open, the ratio is larger, and when they are closed or blinking, the ratio decreases. We implemented the EAR algorithm in Python (v3.10.4) using the *Dlib* (v19.24.6) library [52].

**Blink Frequency**: To measure blink frequency, we also used the EAR algorithm. Referring to previous studies measuring blink frequency [53,54,55,56,57,58], which indicate that 0.25 is an empirically appropriate threshold that effectively balances the risk of missing subtle eye movements (noise) against overcounting blinks, and ensures reliable results across different conditions such as lighting and face angles—we set 0.25 as our criterion for counting blinks.

**Gaze Coordinates**: To collect users’ gaze coordinates, we employed eye trackers capable of monitoring eye movements. Specifically, we used a *Tobii Eye Tracker 5 (Tobii, Stockholm, Sweden)* device, which does not require any direct attachment to the participant. The *Tobii Eye Tracker 5 (Tobii, Stockholm, Sweden)* typically maintains an accuracy of about 30 pixels (1.01°) from the user’s true gaze point across the entire screen [59], and it has been widely used in UX and cognitive studies involving gaze-based metrics [60,61,62,63]. As in previous research, all experiments were conducted on a Full-HD (1920 × 1080) monitor to ensure consistent resolution conditions for all participants. We integrated the *Tobii Game Integration SDK* (v9.0.4.26) within a Unity environment to gather gaze coordinates (for instance, x and y), along with timestamps in milliseconds. These data were used to check whether a users’ gaze coordinate was located inside the blurred-box coordinates of the video. Before the experiment, each participant underwent a personal calibration using the *Tobii Experience* app (v1.69.32600). During calibration, the participants were instructed to keep their bodies and heads stable, moving only their eyes to follow a point on the screen. After the calibration, participants were allowed to move their head and body freely while watching the video during the experiment. These gaze coordinates were used to determine whether the participants looked at the blood or blurred regions within the video clips.

**Figure 1:**
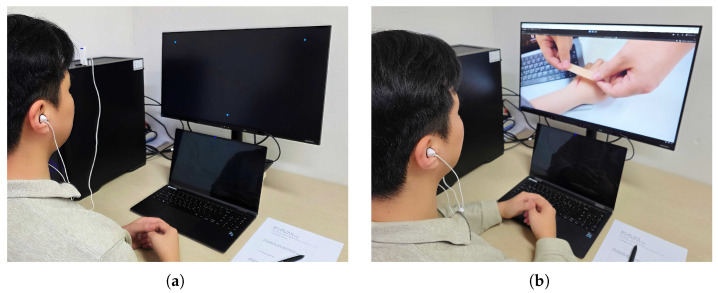
Experimental environment for video clip viewing. (**a**) A participant performing an eye-tracking calibration procedure (an eye tracker is attached to the lower bezel of the monitor). (**b**) A participant watching the experimental video while sensor data (e.g., Eye Openness Ratio and Blink Frequency) are collected.

We applied calibration to three types of sensor data—*Eye Openness Ratio*, *Blink Frequency*, and *Gaze Coordinates*. The *Eye Openness Ratio* and *Blink Frequency*, recorded via a webcam using the Eye Aspect Ratio (EAR), were logged whenever a change in eye movement was detected (see Figure 1). On average, about three data points were captured per 0.1 s, so we used interpolation to normalize the values at 0.1 s intervals. Similarly, the *Gaze Coordinates* from an eye tracker were logged at approximately 30 frames per second, and we employed an averaging interpolation method to align them with the same 0.1 s intervals.

Over the course of about one hour, participants viewed 10 video clips, 5 original versions and 5 blurred to reduce bloody content, and then completed a brief interview after each viewing. Following each video, they rated its gore level on a 7-point Likert scale (1 = not gory at all; 7 = extremely gory), in line with prior research that used a 7-point scale to capture nuanced differences in perceived brutality [64,65]. They also provided a written rationale for their rating and participated in an interview about their viewing experience. To facilitate relative comparisons, the participants were instructed to treat the clip from *Saving Private Ryan (1998)* as having a score of 4, the midpoint of the 7-point scale.

The participants were informed that they could take breaks before or after viewing any video clip, and that they were free to close their eyes or look away from the screen if they felt uncomfortable. They were also assured that there would be no penalty for taking such breaks. All interviews were recorded with the participants’ consent, and upon completing the experiment, each participant received KRW 20,000 (approximately USD 15) as compensation.

## 4. Results

In this section, we used both user survey responses (the level of perceived gore) and sensor data (*Eye Openness Ratio*, *Blink Frequency*, and *Eye Fixation on Blood Regions Using Gaze Coordinates*) to explore the effects of our blurring model by applying statistical analyses. First, we ran a Shapiro–Wilk test to check for normality. Based on those results, we performed either a Wilcoxon signed-rank test (or a Friedman test for comparisons among three or more samples) or a *t*-test (or an ANOVA for three or more samples). All analyses were basically conducted at a 95% confidence level (alpha = 0.05).

We present our findings for RQ1 in Section 4.1, for RQ2 in Section 4.2, Section 4.3 and Section 4.4, and for RQ3 in Section 4.5.

### 4.1. Comparison of Perceived Gore Across Multiple Perspectives

**Figure 2:**
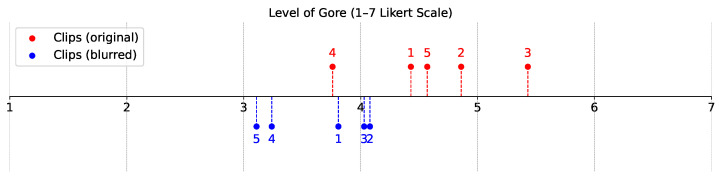
The perceived level of gore.

We examined whether the perceived level of gore differed across the video clips for each version (original vs. blurred). A Friedman test showed statistically significant differences in both versions (p<0.001). A Dunn–Bonferroni post-hoc analysis indicated that for the original version, Clip 4 differed significantly from Clips 1, 2, 3, and 5, whereas for the blurred version, Clip 5 differed significantly from Clips 1, 2, and 3, and Clip 4 differed significantly from Clips 2 and 3. All pairwise comparisons were significant at the 95% confidence level (see Figure 2 and Table 2).

Next, we investigated whether blurring influenced perceived gore. Overall, the participants rated the original versions of the clips as more gory than the blurred versions for all five videos. Statistical tests confirmed significant differences in perceived gore between the two versions of each clip.

We also examined a potential *contrast effect* by comparing two viewing orders: the original-first group (N = 19) and the blurred-first (N = 18) group. Our statistical analysis confirmed significant differences in Clips 1, 2, 3, and 5 for the original-first group, and in Clips 1, 3, 4, and 5 for the blurred-first group. Although the p-values for Clip 4 in the original-first group (p=0.055) and Clip 2 in the blurred-first group (p=0.090) did not reach the conventional 95% significance threshold, they suggest a marginal effect that might be considered meaningful under a less conservative threshold (alpha=0.1) or a one-sided test. Despite these borderline values, the overall pattern indicates that perceived gore was consistently lower in the blurred versions regardless of viewing order.

We analyzed the open-ended responses that the participants provided to explain their perceived gore ratings for each clip. Two authors employed affinity diagramming with open coding to categorize these responses, iterating until they reached consensus. The relevant excerpts were then examined to develop detailed descriptions and illustrative examples of participant behaviors. As a result, we identified six key factors, as shown in Table 3.

These perceived levels of gore—and the differences for each pair of clips—were confirmed by the sensor data analyses presented in the following sections.

### 4.2. Eye Openness Ratio During Gory Video Viewing

We measured each participant’s *Eye Openness Ratio* (based on EAR) at 0.1 s intervals while they were watching the video clips. Because individual EAR values can differ widely, we normalized each value by dividing it by the highest EAR value that a particular participant reached during the clip. As a result, we obtained a ratio ranging from 0 to 1 for every 0.1 s interval. We then averaged these ratios to obtain each participant’s mean *Eye Openness Ratio* for that clip as shown in Table 4. Unfortunately, due to an error in the EAR measurement code, the detailed EAR data for the first 13 participants were compromised. As a result, we excluded those 13 participants from the *Eye Openness Ratio* analysis and used only the remaining 24 participants’ data.

In general, participants tended to keep their eyes more closed when watching the original clips, possibly due to frowning or partially shutting their eyes in response to the graphic bloodshed. We found significant differences in the *Eye Openness Ratio* for Clips 2, 3, and 5, which were rated as relatively gorier than Clips 1 and 4.

We conducted a statistical analysis to determine whether viewing order (original-first vs. blurred-first) influenced participants’ *Eye Openness Ratio*. Among those who watched the original version first, the results generally mirrored the overall trends: Clips 3 and 5, which were rated as relatively gorier, showed significant differences in eye openness between the original and blurred versions. For Clip 2, which was identified as the second-goriest, the *p*-value (0.054) was slightly above the threshold, so it was not deemed significant. In contrast, participants who watched the blurred version first did not exhibit statistically significant differences for all clips. However, these findings require cautious interpretation due to the smaller sample sizes in each group, a consequence of losing data from 13 participants in this measurement.

Our qualitative interview analysis corroborated these findings. Some participants reported that they tended to close their eyes more often when watching gorier scenes. For example, P30 and P1, referring to Clip 3 (original), commented the following:

*“The scene was so graphic that it was hard to keep my eyes open.”*

*“I felt like it was an actual nose surgery, so I couldn’t stop grimacing the whole time. And of course, since I’ve got a nose too, my imagination was all the more easily triggered, I guess.”*

On the other hand, some participants indicated that they closed their eyes less frequently when viewing blurred versions of the clips. For instance, P11 commented the following on Clip 1 (blurred):

*“Now that the bloody area on the face is covered (by blurring), it feels like a lot of the gruesome elements are gone, so I definitely noticed myself grimacing less frequently.”*

### 4.3. Blink Frequency

We measured each participant’s *Blink Frequency* (using EAR) at 0.1 s intervals while they watched the video clips, adopting a threshold of 0.25 to identify blinks based on prior studies. Table 5 presents the mean absolute blink counts of the participants for each clip. Because these counts reflect total occurrences, the average blink count tends to be higher for longer clips. For reference, a normal blink rate is approximately 17 times per minute [66].

We observed that the participants generally blinked more when watching the original versions compared to the blurred versions, except for Clip 4, which was rated the least gory. Statistical analysis confirmed significant differences in *Blink Frequency* for the other four clips.

To further investigate potential order effects, we split participants into two groups based on which version (original or blurred) they viewed first. Among those who started with the original version, no clip showed a statistically significant difference in blink frequency between the original and blurred versions. Conversely, participants who began with the blurred version exhibited significant differences in *Blink Frequency* for Clips 2, 3, and 5, which were comparatively more gory.

### 4.4. Analyzing Eye Fixations on Blood Regions Using Gaze Coordinates

We measured each participant’s *Eye Fixations* on blood regions using *gaze coordinates* at 0.1 s intervals during video viewing. Table 6 summarizes the average fixation duration for each clip. To normalize these data, we calculated the proportion of 0.1 s intervals in which participants fixated on blood (or blurred) regions, relative to the total number of frames containing such regions in each clip. Thus, a value of 50 would indicate that a participant fixated on the relevant frames 50% of the time.

We observed that participants tended to fixate longer on the blood area when the gore level was lower. Statistical analysis revealed significant differences in *Eye Fixations* for Clips 2, 3, and 5, which were rated more gory than Clips 1 and 4. The result confirmed that the participants spent more time looking at the blurred-box area in the blurred version, suggesting they avoided this region when the blood was fully visible.

We also explored whether viewing order (original first vs. blurred first) affected participants’ *Eye Fixations*. In the blurred-first group, Clips 1, 2, and 3 showed significantly longer fixations on the blood area in the blurred version. For Clips 2 and 3—the most gory clips—this outcome is consistent with the findings for the entire participant sample. By contrast, in the original-first group, only Clip 1 exhibited a significant difference, with participants devoting more fixation time to the blood area in the original version. Two possible reasons may account for this discrepancy. First, a “learning effect” could have occurred, since many participants were already familiar with the movie *Parasite* (from which Clip 1 was taken). This film is extremely famous in South Korea due to winning four Academy Awards in 2020, and 30 participants reported having seen it before this experiment. Second, Clip 1 depicts an outdoor party with many people and diverse objects, unlike the relatively contained settings of the other clips. Consequently, there were multiple elements that might draw viewers’ attention, thus reducing the relative focus on the blood area.

Our interview findings also complemented the *Eye Fixations* results. For instance, when asked about Clip 1, P15 noted the following:

*“I had already seen this movie before, which might have influenced me. Also, it has a lot of elements that are distracting beyond just the gory parts.”*

Meanwhile, P13 and P32 described how the blur helped them watch the blood area in Clips 2 and 5, respectively:

*“Maybe because it was blurred, it felt less tiring than the original version. The scene of putting one’s hand in the belly to find the aorta was blurred, which made it more comfortable to watch.”*

*“Because it was blurred, I didn’t think it was so gory that I couldn’t keep watching.”*

These comments underscore how blurring can reduce discomfort when viewing blood, making graphic scenes more tolerable for participants.

### 4.5. Blood/Injury Phobia Questionnaire

We employed the Multidimensional Blood/Injury Phobia Inventory (MBPI) [46] to assess participants’ blood- and injury-related phobias. Specifically, we selected three subscales focusing on blood-related fears from the MBPI:

- *Blood-Self* (four items): evaluates fears associated with one’s own blood.
- *Injury* (six items): captures apprehension about sustaining injuries and the negative emotional responses that accompany them.
- *Blood/Injury-Others* (five items): measures fears triggered by witnessing others’ blood or injuries.

Each item was rated on a four-point Likert scale, ranging from 1 (very slightly or not at all) to 4 (extremely). For the *Blood-Self* subscale, the average score was 1.66 (SD = 0.55). The *Injury* subscale had an average score of 2.86 (SD = 0.63), and the *Blood/Injury-Others* subscale showed an average score of 2.16 (SD = 0.68). Overall, participants’ *Blood-Self* scores were relatively low, whereas their *Injury* scores were comparatively high.

**Figure 3:**
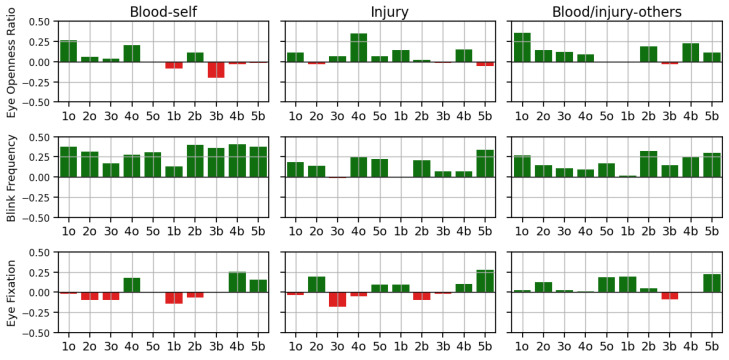
The perceived level of gore (#o: original version of Clip #, #b: blurred version of Clip #).

We then examined how participants’ MBPI subscales related to their sensor data—*Eye Openness Ratio*, *Blink Frequency*, and *Eye Fixations*—using Pearson’s correlation (see Figure 3). Notably, *Blink Frequency* exhibited stronger correlations than the other measures. In particular, we found that *Blink Frequency* and *Blood-Self* showed a moderate yet statistically meaningful association. Although these correlations are not extremely high, they still suggest a significant relationship worthy of further investigation.

## 5. Discussion

### 5.1. Effect of Segmentation-Based Blood Blurring on Perceived Gore (RQ1)

We first investigated the extent to which segmentation-based blood blurring reduces viewers’ perceived gore. Our findings indicate that this approach significantly lowered perceived gore across all five video clips. Even when separating participants into original-first or blurred-first viewing groups, the blurred versions consistently produced lower gore ratings in most clips.

To deepen our understanding of these results, we identified multiple elements influencing perceived gore, including *Bleeding*, *Severity of Injury*, *Violent Behavior*, *Victim’s Reaction*, *Realism*, and *Sound*. Although our blurring technique did not address non-blood elements such as victim reactions or sound, we found that obscuring blood regions substantially reduced participants’ perceived gore. These observations align with prior research highlighting the importance of blood color, sound effects, and the overall realism of injury [36], as well as the pivotal role blood quantity plays in provoking aggressive thoughts and physiological arousal [11]. Future work could consider designing adaptive blurring strategies that vary according to the severity or volume of blood, potentially achieving greater reductions in perceived gore. Moreover, other factors that can heighten the sense of brutality—such as blood color, verbal tone, and lighting—have also been proposed [67], suggesting that a more comprehensive content moderation system might combine multiple forms of obfuscation or sensory modifications to fully address the diverse elements that contribute to perceived gore.

### 5.2. Physiological Reactions During Gory Video Viewing (RQ2)

Our study revealed that physiological reactions (i.e., eye openness ratio and blink frequency) differ significantly depending on the level of gore in the video clips. Most previous research compares physiological responses to highly disturbing content or entirely neutral material. We addressed this gap by examining user responses to the same clips in both blurred and unblurred formats.

When viewing gorier clips, the participants tended to close their eyes more and blink more. In addition, they looked at the blood area within the blurred versions of clips for a longer period of time compared to in the original versions. In particular, the differences were clearly observed for Clips 2 and 3, which represent the two highest levels of gore. However, no significant differences were observed in Clip 4, which represents the lowest level of gore, although it describes a gory scene rather than a natural or normal one.

The physiological responses reported in our study are in line with previous findings on reactions to violent, frightening, or disturbing stimuli. Researchers have noted that individuals display heightened facial tension—such as eye squeezing—when confronted with fear-inducing content, and relax their facial muscles when viewing neutral or nonthreatening material [25,29]. Increased blink frequency has also been observed as part of the startle response when the body recognizes and responds to perceived threats, with aggressive or shocking imagery further amplifying this reflex [31]. In addition, extreme gore can trigger discomfort and emotional avoidance, resulting in higher spontaneous blink rates (SBRs) [24]. These diverse mechanisms—ranging from facial muscle tension to reflexive blinking—underscore how different aspects of harmful content (e.g., severity, aggression, disgust) can elicit a range of involuntary reactions aimed at regulating emotional and attentional states.

### 5.3. Blurring and Gaze Engagement: The Role of Curiosity (RQ2)

Our study revealed that eye gaze patterns vary significantly with the level of gore in the video clips. Notably, participants fixated on blood regions longer when these areas were blurred rather than left unaltered—an outcome that contrasts with prior findings suggesting that viewers typically exhibit reduced fixation times and a quicker tendency to avoid frightening or disturbing stimuli [25,28]. This result indicates that blurring may mitigate viewers’ instinctive avoidance of gory scenes, allowing them to stay more engaged and gather additional contextual information.

Such a phenomenon can be interpreted through the lens of *Information Gap Theory* [68] and *Cognitive Curiosity* [69]. According to these theories, curiosity is triggered when individuals recognize a gap between what they already know and what they desire to know. In the context of gory scenes, viewers may want to understand the full narrative but feel deterred by explicit depictions of blood, which can prompt them to look away and lose crucial context. Visual avoidance is a common emotion regulation strategy aimed at reducing negative responses such as disgust [70] or fear [71]; by turning away, viewers attempt to down-regulate these negative feelings via behavioral responses [72].

By applying segmentation-based blurring, our approach selectively obscures only the most graphic elements while preserving other visual information. This strategy reduces emotional or attentional barriers, enabling viewers to satisfy their curiosity about the scene without the heightened discomfort that can stem from explicit imagery. As a result, blurring potentially closes the *information gap*, encouraging viewers to maintain visual engagement and better understand the scene’s context.

### 5.4. Contrast and Order Effects in Gory Content Viewing (RQ2)

Our findings align with prior research on *contrast* and *order effects*, demonstrating that the perceived intensity of a new stimulus is influenced by the severity of previously viewed content. Participants who first watched the unblurred, highly gory clip subsequently rated the blurred version as less disturbing, illustrating a *contrast effect* in which an intense stimulus overshadows a milder one that follows [73,74,75,76]. Conversely, those who began with the blurred clip tended to perceive the unblurred one as more horrifying, reflecting an *order effect* in which an initial, less intense experience increases expectations for the next [77,78].

In practical terms, continuous viewing on platforms like YouTube Shorts, TikTok, or Reels may magnify negative reactions when a sudden transition occurs from mildly to extremely violent content, or normalize violence if several gory clips are watched consecutively. Our results highlight the need for careful consideration of such contrast effects and ordering in designing streaming services that present diverse, rapidly shifting videos.

### 5.5. Empathy and Physiological Responses to Gory Scenes (RQ3)

To examine how an individual’s fear level (i.e., blood- or injury-related phobias) is associated with the viewing experience of gory videos, we measured the participants’ blood- and injury-related phobias using MBPI subscales [46], *Blood-Self*, *Injury*, and *Blood/Injury-Others*, and investigated the relation with their sensor data, *Eye Openness Ratio*, *Blink Frequency*, and *Eye Fixations*, using Pearson’s correlation. We found that *Blood-Self* exhibited moderate correlations with *Blink Frequency*, meaning that an individual who rated himself with high blood-self blinked more when viewing, in particular, clips with relatively higher levels of gore (i.e., Clips 2, 3, and 5). It is notable that the physiological reaction of *Blink Frequency* showed a clearer correlation with *Blood-Self* than *Blood-Other*, even though they watched someone else’s bleeding in the clips, not their own bleeding.

One possible explanation involves *empathy*, which refers to the capacity to recognize and share another person’s feelings or experiences. This concept includes pain empathy [79], physical empathy [80], cognitive empathy [81], sensory sharing [82], and vicarious pain [83]. For instance, one study demonstrated that when participants were shown images of individuals experiencing various forms of physical pain, they reported empathic distress similar to the emotions associated with feeling pain themselves [84]. Such empathic responses can promote greater immersion, yet viewers may need to guard against overidentification—particularly in cases of extreme gore—in order to recognize the fictional context and prevent excessively negative viewing experiences [80].

In this regard, our blurring technique may serve as a protective mechanism by lowering the intensity of graphic imagery. For example, repeated or extended exposure to violent, lengthy videos has been shown to desensitize viewers to real-life violence [85,86] and others’ suffering, ultimately reducing prosocial behavior [87]. Moreover, young people frequently exposed to violent media can exhibit increased physical aggression and diminished empathy [88]. By mitigating the visual impact of blood and gore, our approach helps prevent viewers from becoming overly distressed during violent or bloody scenes.

### 5.6. Design Implications

Based on our findings, we propose two design implications for online video streaming services aimed at enhancing users’ viewing experiences. These suggestions illustrate how segment-based blood blurring and responsive moderation could be practically implemented in light of our study’s results.

#### 5.6.1. Implication for the Segment-Based Blood Blurring on Video Streaming Service

**Figure 4:**
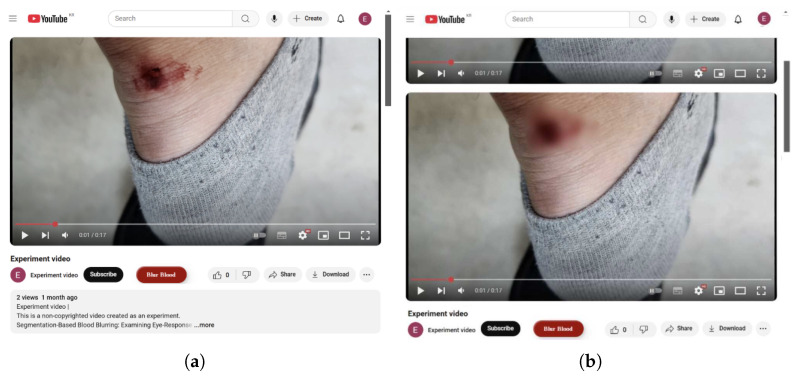
A Chrome browser extension enabling segment-based blood blurring on YouTube. (**a**) A standard YouTube video page with the “Blur Blood” button added below the title. (**b**) The page after generating a blurred version of the video, displayed below the original player.

To explore real-world applicability, we developed a Chrome browser extension that enables users to watch a blood-blurred version of any YouTube video (see Figure 4). When a user visits a YouTube video page, the extension automatically adds a “Blur Blood” button beneath the video title. If the user anticipates disturbing or violent content, clicking the button prompts the extension to download the video, using the *yt-dlp (v2024.10.22)* library [89], to a server where it is processed through our YOLO-based blurring model. The resulting blurred video is then inserted below the original video player, allowing viewers to watch it in a more comfortable manner while still following the narrative context.

This prototype demonstrates the technical feasibility of offering a blurred viewing experience for users who prefer to avoid graphic imagery. Future work can focus on user-centered refinements, such as giving viewers control over the blur intensity or offering additional personalization options. These enhancements could promote a more inclusive viewing environment, accommodating individual differences in gore tolerance and further reducing unintentional exposure to violent or bloody scenes.

#### 5.6.2. Camera-Based Detection of Discomfort and Responsive Blurring on Smartphones

Our study showed that participants’ physiological and gaze responses (e.g., eye openness ratio and blink frequency) varied significantly according to the clip’s gore level. This finding suggests the possibility of dynamically predicting a viewer’s discomfort through camera-based sensors (e.g., built-in webcams or eye trackers) and applying a corresponding level of blur. Many users watch online videos on mobile devices equipped with front-facing cameras, which could facilitate such real-time monitoring. A responsive blur system could, for instance, automatically increase the blurring intensity when it detects that the viewer’s discomfort is rising, helping them remain engaged with the content without feeling excessively disturbed.

However, for this suggested idea, privacy concerns must be carefully addressed, particularly regarding the continuous monitoring of users’ facial expressions or gaze. If implemented responsibly, this adaptive approach could empower viewers to experience less distressing but still informative versions of violent or gory videos. It would also open avenues for personalized moderation strategies that prioritize user comfort and well-being.

### 5.7. Limitation and Future Work

*Sample size and composition*: One limitation of this study involves the sample’s size and composition. The overall number of participants was relatively small (N = 37), which becomes more pronounced when comparing the original-first and blurred-first groups. Furthermore, all participants were young adults from South Korea, and potential gender- and cultural-related differences were not examined. Specifically, previous research has noted that men and women may differ in their responses to violent or graphic media [34,90], suggesting a need for broader demographic sampling in future work to enhance generalizability. Additionally, for the *Eye Openness Ratio* analysis, 13 participants had to be excluded due to a coding error that invalidated their EAR measurements. Although we compared demographics and other key measures for the excluded participants versus the remaining 24 and found no significant differences, the final subset was smaller than intended. This reduction potentially limits the statistical power and scope of our findings related to eye openness. Future studies should employ more robust methods for data collection and validation to minimize such exclusions, as well as explore a wider demographic range to improve representativeness.

*Familiarity with the stimulus clips*: In addition, we did not account for participants’ prior familiarity with the video materials. Notably, 30 out of the 37 participants had already watched the film *Parasite* (Clip 1), whereas very few participants had seen any of the other four clips beforehand. This familiarity may have influenced how individuals perceived the gore or engaged with the content. Future research should therefore incorporate measures of prior viewing experience, especially for well-known films, to better understand how familiarity mediates responses to graphic or blurred content.

*Differences in clip durations*: We also acknowledge that the selected clips differ in runtime. Our priority was to preserve each clip’s natural context and flow, rather than artificially trimming or extending the footage to achieve a uniform length. Although this approach strengthened ecological validity, it means runtime consistency was not strictly controlled. We employed a repeated-measures design so that each participant served as their own control for both the original and blurred versions of the same clip. However, future studies may wish to investigate systematically matching or adjusting clip lengths, while retaining essential context, to further clarify how viewing time intersects with physiological responses and perceived gore.

*Evaluating the blurring model and future improvements*: The accuracy of the blurring process itself may have influenced viewer reactions. Although participants generally felt the blurring was well applied, numerical assessments revealed that the method was not perfectly accurate. In addition, we did not measure how much segmentation-based blurring helped viewers understand contextual details. The effectiveness of a segmentation-based approach, compared to box-based blurring, was not formally evaluated, even though participant feedback (e.g., “I could simply recognize ‘Oh, there’s rebar there!’ without dwelling on the graphic aspects”, from P29) suggests that preserving contextual information can be advantageous. Under a box-based method, the entire area might have been obscured, hindering the recognition of crucial details. In future work, researchers could refine or develop more advanced algorithms to optimize both detection precision and viewer comfort, ensuring that observed outcomes truly result from the blurring intervention rather than technology-related shortcomings. We also believe that systematically examining the added benefits of segmentation-based blurring, particularly its ability to mitigate perceived gore while preserving context, is vital for enhancing the overall viewing experience of gory videos and expanding the contributions of this research.

*Accessibility concerns*: In our study, we focused on blurring blood. Although individuals with color insensitivity may not perceive redness as vividly, we believe that blurring can still reduce perceived gore by de-emphasizing blood flow, since the absence of full color perception does not necessarily eliminate awareness of the amount or motion of bleeding. However, its effectiveness may differ for color-insensitive viewers. To ensure our system benefits a broader range of users, future work should address accessibility concerns, similar to how some webtoon designs are adapted for color-insensitive readers [91].

## 6. Conclusions

In this study, we examined three research questions: (1) the extent to which segmentation-based blood blurring reduces viewers’ perceived gore, (2) how different levels of gore influence the viewing experience (including eye-based physiological responses and gaze behavior), and (3) in what ways an individual’s fear level correlates with watching gory videos. To achieve these objectives, we developed and evaluated a segmentation-based blood-blurring model designed to reduce the perceived gore of violent scenes without entirely removing important contextual information. Our experiment, involving 37 participants, revealed that partial blurring significantly lowered viewers’ perceived gore, particularly for clips with higher levels of brutality. Moreover, physiological and eye-gaze data indicated distinct patterns of eye openness, blink frequency, and fixation behaviors that corresponded to the intensity of gory content. Individual differences, including self-reported fear of blood, further influenced responses such as blink rate, demonstrating the applicability of personalized content moderation. By combining user-centered experimentation with deep learning-driven video processing, this work offers practical insights for designing and implementing more nuanced content-filtering systems. Future research may explore adaptive blurring that responds to real-time physiological signals, investigate additional factors (e.g., audio cues, context of violence), and further refine approaches to safeguard vulnerable viewers while preserving narrative coherence.

## Figures and Tables

**Table 2:** Perceived gore ratings by clip and version.

Clip	Version	Total (N = 37)		Original First (N = 19)		Blurred First (N = 18)	
		Mean (SD)	Statistic	Mean (SD)	Statistic	Mean (SD)	Statistic
Clip 1	Original	4.43 (1.53)	W=23.0	4.32 (1.63)	W=0.0	4.56 (1.46)	W=11.0
	Blurred	3.81 (1.54)	*** p<0.001	3.58 (1.57)	*** p<0.001	4.06 (1.51)	* p=0.033
Clip 2	Original	4.86 (1.20)	W=55.0	4.68 (1.20)	W=4.5	5.06 (1.21)	W=28.0
	Blurred	4.08 (1.27)	*** p<0.001	3.53 (1.30)	** p=0.001	4.67 (0.97)	p=0.090
Clip 3	Original	5.43 (1.60)	W=19.5	4.74 (1.72)	W=6.0	6.17 (1.09)	W=4.0
	Blurred	4.03 (2.10)	*** p<0.001	2.95 (2.12)	** p=0.003	5.17 (1.38)	** p=0.003
Clip 4	Original	3.76 (1.34)	W=47.5	3.32 (1.24)	t=2.051	4.22 (1.30)	W=4.0
	Blurred	3.24 (1.40)	** p=0.006	2.68 (1.20)	p=0.055	3.83 (1.38)	* p=0.035
Clip 5	Original	4.57 (1.34)	W=42.5	4.68 (1.45)	W=16.0	4.44 (1.24)	t=4.914
	Blurred	3.11 (1.39)	*** p<0.001	3.21 (1.65)	** p=0.002	3.00 (1.08)	*** p<0.001

Note. Asterisks denote statistical significance (* p<0.05, ** p<0.01, *** p<0.001), and significant results are shown in **bold**.

**Table 3:** Factors influencing perceived gore.

Factor	Definition
Bleeding	Scenes in which blood is actively spurting or flowing (e.g., a wound constantly gushing blood).
	*“Blood was coming out more here than in any other video I’ve watched, so it was really tough to handle.” (P6)*
	*“Seeing blood spewing made it feel more severe than the previous clip. I focused on the gushing blood and judged it to be very gruesome.”* (P4)
Severity of Injury	Scenes in which the injury is visibly severe (e.g., bone exposed, large open wounds).
	*“The wound was around the neck area, possibly fatal, and the back injury was so deep you could almost see the bone. It felt very disturbing.”* (P5)
	*“Seeing such a deep, detailed wound was extremely unpleasant for me.”* (P6)
Violent Behavior	Cruel or aggressive actions carried out by someone (e.g., stabbing with a knife).
	*“When there was a blur, I thought they were treating the wound, but they were actually sticking fingers in it. That was really gross.”* (P6)
	*“There was a scene showing direct physical harm with a knife, which I found brutal.”* (P13)
Victim’s Reaction	How the injured individual responds (e.g., expressions of pain, screams, visible distress).
	*“When the person was stabbed, their reaction and the actor’s performance made it feel more brutal.”* (P8)
	*“Seeing someone in severe pain and anguish was much more disturbing, so that part made it extra gruesome.”* (P24)
Realism	The degree of realistic detail in a scene (e.g., fully depicted surgical procedures or lifelike injuries).
	*“It was a surgical scene, so it seemed more realistic than usual.”* (P15)
	*“Unlike other videos, this one felt so real that it intensified the gore for me.”* (P26)
Sound	Auditory cues that enhance the impact of gore or violence (e.g., the sound of a knife piercing flesh).
	*“When the hand was stabbed, the sound was so vivid it felt really unsettling.”* (P37)
	*“Hearing the gunfire made the scene more horrific for me.”* (P18)

**Table 4:** Eye Openness Ratio by clip and version.

Clip	Version	Total (N = 24)		Original First (N = 12)		Blurred First (N = 12)	
		Mean (SD)	Statistic	Mean (SD)	Statistic	Mean (SD)	Statistic
Clip 1	Original	81.61 (6.53)	W=121.0	82.07 (4.62)	W=24.0	81.15 (8.21)	t=0.430
	Blurred	81.76 (7.56)	p=0.874	79.42 (8.56)	p=0.465	84.11 (5.86)	p=0.676
Clip 2	Original	78.82 (6.90)	t=−2.867	78.02 (5.63)	W=11.0	79.62 (8.16)	t=−1.692
	Blurred	81.10 (6.46)	** p=0.009	80.16 (5.19)	p=0.054	82.03 (7.65)	p=0.122
Clip 3	Original	77.09 (8.19)	W=53.0	77.12 (6.90)	W=6.0	77.05 (9.64)	W=21.0
	Blurred	81.37 (9.11)	* p=0.016	78.69 (9.19)	* p=0.014	84.05 (8.57)	p=0.320
Clip 4	Original	81.46 (6.21)	W=112.0	80.51 (7.12)	W=15.0	82.41 (5.29)	W=30.0
	Blurred	82.48 (5.41)	p=0.656	81.32 (4.97)	p=0.123	83.64 (5.80)	p=0.831
Clip 5	Original	79.15 (6.88)	W=52.0	76.74 (7.19)	t=−2.462	81.56 (5.88)	W=17.0
	Blurred	82.23 (6.43)	* p=0.014	80.40 (7.05)	* p=0.034	84.06 (5.45)	p=0.175

Note. Asterisks denote statistical significance (* p<0.05, ** p<0.01, *** p<0.001), and significant results are shown in **bold**.

**Table 5:** Blink Frequency by clip and version.

Clip	Version	Total (N = 37)		Original First (N = 19)		Blurred First (N = 18)	
		Mean (SD)	Statistic	Mean (SD)	Statistic	Mean (SD)	Statistic
Clip 1	Original	56.46 (48.72)	W=218.5	62.68 (53.85)	W=49.0	49.89 (43.22)	W=61.5
	Blurred	44.51 (46.98)	* p=0.046	43.16 (33.30)	p=0.066	45.94 (59.13)	p=0.325
Clip 2	Original	91.16 (78.31)	W=181.0	105.05 (80.02)	W=65.0	76.50 (75.93)	W=25.0
	Blurred	77.11 (69.24)	** p=0.009	96.74 (74.07)	p=0.241	56.39 (58.83)	** p=0.007
Clip 3	Original	20.32 (19.11)	W=96.5	18.95 (12.50)	W=45.5	21.78 (24.58)	W=4.0
	Blurred	14.78 (14.74)	*** p<0.001	16.16 (13.67)	p=0.142	13.33 (16.07)	*** p<0.001
Clip 4	Original	40.86 (45.88)	W=327.5	49.63 (58.29)	W=66.5	31.61 (26.17)	W=67.5
	Blurred	37.05 (36.01)	p=0.931	47.26 (39.57)	p=0.408	26.28 (29.14)	p=0.468
Clip 5	Original	71.76 (69.91)	W=174.0	83.95 (80.32)	W=60.0	58.89 (56.39)	W=31.0
	Blurred	53.73 (58.70)	* p=0.012	71.95 (74.42)	p=0.169	34.50 (25.91)	* p=0.031

Note. Asterisks denote statistical significance (* p<0.05, ** p<0.01, *** p<0.001), and significant results are shown in **bold**.

**Table 6:** Eye fixation ratio on blood regions by clip and version.

Clip	Version	Total (N = 37)		Original First (N = 19)		Blurred First (N = 18)	
		Mean (SD)	Statistic	Mean (SD)	Statistic	Mean (SD)	Statistic
Clip 1	Original	34.14 (8.46)	t=0.670	38.53 (6.65)	t=3.358	29.51 (7.79)	t=2.751
	Blurred	32.99 (8.09)	p=0.507	31.42 (9.48)	** p=0.004	34.64 (6.15)	* p=0.014
Clip 2	Original	51.30 (10.97)	W=205.0	55.32 (4.89)	t=−0.256	47.07 (1.88)	W=31.0
	Blurred	55.28 (6.34)	* p=0.044	55.73 (6.29)	p=0.800	54.80 (6.56)	* p=0.016
Clip 3	Original	47.58 (18.78)	W=191.0	51.68 (16.53)	W=68.0	43.25 (20.47)	W=22.5
	Blurred	56.55 (18.75)	* p=0.026	55.84 (22.12)	p=0.446	57.31 (15.01)	** p=0.004
Clip 4	Original	58.14 (11.12)	W=286.0	58.66 (9.94)	t=0.321	57.58 (12.52)	t=−0.784
	Blurred	58.85 (12.54)	p=0.844	57.74 (14.18)	p=0.752	60.01 (10.85)	p=0.444
Clip 5	Original	41.20 (8.03)	t=−2.713	40.95 (8.64)	W=52.0	41.47 (7.59)	t=−1.946
	Blurred	45.04 (8.94)	* p=0.010	44.86 (8.60)	p=0.087	45.24 (9.86)	p=0.068

Note. Asterisks denote statistical significance (* p<0.05, ** p<0.01, *** p<0.001), and significant results are shown in **bold**.

## Data Availability

Our dataset is available at https://doi.org/10.5281/zenodo.14869369 and includes participants’ perceived gore ratings, eye openness ratio, blink frequency, eye fixation metrics, and MBPI subscale responses.
